# Critical Illness-Related Alterations in Plasma Phosphatidylcholine Species Reveal Selective Induction of PC 32:0

**DOI:** 10.3390/ijms27156858

**Published:** 2026-07-30

**Authors:** Patricia Mester, Vlad Pavel, Stephan Schmid, Marcus Höring, Gerhard Liebisch, Martina Müller, Christa Buechler

**Affiliations:** 1 Department of Internal Medicine I, Gastroenterology, Hepatology, Endocrinology, Rheumatology, and Infectious Diseases, University Hospital Regensburg, 93053 Regensburg, Germany; patricia.mester@klinik.uni-regensburg.de (P.M.); vlad.pavel@klinik.uni-regensburg.de (V.P.); stephan.schmid@klinik.uni-regensburg.de (S.S.); martina.mueller-schilling@klinik.uni-regensburg.de (M.M.); 2 Institute of Clinical Chemistry and Laboratory Medicine, University Hospital Regensburg, 93053 Regensburg, Germany; marcus.hoering@klinik.uni-regensburg.de (M.H.); gerhard.liebisch@klinik.uni-regensburg.de (G.L.)

**Keywords:** sepsis, phosphatidylcholine, liver cirrhosis, COVID-19

## Abstract

Circulating lipoprotein concentrations decrease in critical illness, suggesting that plasma phosphatidylcholine (PC) levels may also decline. However, published data on individual PC species in critical illness are conflicting, and underlying liver cirrhosis may substantially influence systemic lipid profiles. We therefore examined the impact of systemic inflammatory response syndrome (SIRS)/sepsis, with and without liver cirrhosis, on circulating PC species. We quantified 21 plasma PC species in 160 patients with SIRS, sepsis, or septic shock, including 31 patients with liver cirrhosis. PC profiles were compared with those of healthy controls and across clinically relevant subgroups. In patients with SIRS/sepsis without liver cirrhosis, 15 PC species were reduced compared with healthy controls, whereas PC 32:0 was increased. In patients with SIRS/sepsis and concomitant liver cirrhosis, 12 PC species were lower than in septic patients without cirrhosis, while PC 32:0 was again increased. Because a proportion of circulating PC is derived from phosphatidylethanolamine (PE), we explored whether altered PE-to-PC conversion could account for these changes; however, the observed pattern did not support this mechanism. No significant differences in PC species were observed between survivors and non-survivors. In summary, critical illness is associated with a broad reduction in circulating PC species, and this decrease is accentuated by coexisting liver cirrhosis. In contrast, PC 32:0 is consistently increased in both SIRS/sepsis and SIRS/sepsis with cirrhosis, suggesting a distinct biological role that warrants further investigation.

## 1. Introduction

Sepsis, defined as life-threatening organ dysfunction resulting from a dysregulated host response to infection, remains a major problem in intensive care medicine owing to its high mortality and persistent long-term consequences [1,2,3]. Among the mechanisms involved, lipid metabolism and lipid mediators have received increasing attention. Beyond their role as membrane constituents, lipids modulate immune and inflammatory pathways, promoting pathogen clearance while also limiting excessive tissue injury. Accordingly, the balance between pro-inflammatory and pro-resolving lipid signaling may be an important determinant of sepsis progression and outcome [4,5,6].

Phosphatidylcholine (PC) is the dominant glycerophospholipid in mammalian cell membranes, accounting for approximately 40–50% of total phospholipids. This high abundance underscores its central structural and functional importance for membrane integrity [7]. The structural diversity of the fatty acid residues bound to the PC molecule, as well as to other lipids, is remarkable. Both chain length and degree of saturation vary considerably, and this variability largely determines the biophysical properties of the cell membrane, in particular fluidity, curvature, and lateral organization. Through these physicochemical parameters, PC regulates crucial membrane-associated processes, including the spatial organization of receptors, the formation of functional membrane domains, and the efficiency of intracellular signal transduction [7,8].

Intraperitoneal injection of polyunsaturated PC protected from inflammation and was effective against lipopolysaccharide-induced systemic inflammation and multi-organ injury in a rodent model [9]. Group II secretory phospholipase A_2_, which hydrolyzes PC to lysophosphatidylcholine, is upregulated in sepsis [10]. These studies suggest that PC species are depleted in inflammatory diseases. Indeed, a marked alteration in the plasma PC profile has been observed in patients with sepsis. However, elevated concentrations of certain species were observed, including PC 15:0/18:2, PC 16:0/18:2, PC 16:0/18:1, and PC 16:0/20:5. In contrast, concentrations of other species, including PC 16:0/20:3 and PC 16:0/20:1, decreased compared to healthy controls [11]. A comprehensive evaluation revealed that patients with sepsis exhibited elevated total PC concentrations [11]. This study included 20 patients with sepsis and 20 healthy controls, which is a limitation of this analysis [11]. In addition, a comparison between patients with systemic inflammatory response syndrome (SIRS) and sepsis showed higher levels of saturated and monounsaturated PCs in sepsis [12]. The presence of certain PC molecules, such as PC 18:2/20:3 and PC 20:0/20:3, was diminished in both sepsis and septic shock cases [13]. The levels of PC 16:0/20:2 and PC 18:0/20:3 were particularly noteworthy, with decreases observed in sepsis and further reductions in septic shock [13]. As Lodge et al. included only two of 152 patients with chronic liver disease [13], this suggests that specific PC species are related to disease severity in critical illness independent of liver disease.

PC, administered either by injection or orally, has also been shown to improve liver health in experimental models [14,15]. In patients with metabolic liver disease, circulating levels of polyunsaturated PCs were inversely associated with markers of liver injury [16], suggesting a protective function of these lipids.

Liver cirrhosis is a major risk factor for infection and is associated with increased mortality [17,18]. Liver cirrhosis is usually associated with lower levels of various lipids, reflecting impaired liver function [19]. Indeed, the presence of PC species containing polyunsaturated fatty acids was significantly reduced in cirrhosis [20,21]. As McPhail et al. demonstrate in their study, certain PC species have been linked to the decompensation of cirrhosis [22]. Moreover, these species have been shown to be further diminished in patients who do not survive [22].

To our knowledge, it has not been investigated whether levels of unsaturated PC species decline further in patients with both liver cirrhosis and severe inflammatory disease than in those with SIRS or sepsis alone, or whether these changes are associated with disease severity and clinical outcomes. Interestingly, patients with chronic hepatitis Crelated cirrhosis have been reported to exhibit lower levels of most PC species and elevated serum levels of PC 30:0 and PC 32:0 [20]. The mechanisms underlying the increased abundance of these saturated PC species and the decline of unsaturated PC species remain unknown, and their relevance in critically ill patients with liver cirrhosis requires further investigation.

A reduced hepatic PC-to-phosphatidylethanolamine (PC/PE) ratio has been associated with impaired lipid secretion and liver injury in patients with chronic liver disease [23]. Whether the serum PC/PE ratio also declines in patients with cirrhosis or in critical illness, which is frequently associated with liver dysfunction [24]—or in patients with both conditions remains unclear.

In the liver, PC is synthesized via two central pathways that contribute to total production to varying degrees: Approximately 30% of hepatic PC is produced via the phosphatidylethanolamine N-methyltransferase (PEMT) pathway, while around 70% is synthesized via the cytidine diphosphate (CDP)–choline pathway [25]. In patients with acute respiratory distress syndrome, PC flux through the CDP–choline pathway is increased, whereas flux through the phosphatidylethanolamine N-methyltransferase (PEMT) pathway is reduced. However, phosphatidylethanolamine (PE) levels were not analyzed in parallel, precluding an assessment of how alterations in PC synthesis affected the PC/PE ratio in blood [26].

PCs are important for liver homeostasis and have been linked to sepsis severity [13,23]. The hepatic PC/PE ratio is essential for liver health. Yet, chronic liver disease and cirrhosis have been insufficiently represented in previous studies [12,13], despite evidence that PC regulation in cirrhosis and sepsis is species-specific [11,13]. Liver cirrhosis is a major risk factor for severe infections and is associated with increased mortality [17,27,28]. Although profound disturbances in lipid metabolism have been described in both cirrhosis and sepsis [4,19,29], their contribution to disease severity and clinical outcome in patients with sepsis and underlying cirrhosis remains poorly understood. We hypothesized that circulating polyunsaturated PC species decline in sepsis and are further reduced in patients with both sepsis and cirrhosis, making them potential diagnostic and, possibly, prognostic biomarkers in this patient population.

This study quantified 21 plasma PC species in healthy controls and patients with SIRS/sepsis to define critical illness-associated PC remodeling and to assess the modifying effect of underlying liver cirrhosis. Additional analyses addressed pancreatitis, cholangiosepsis, and SARS-CoV-2-associated sepsis, which may differentially alter lipid metabolism in critical illness [30].

## 2. Results

### 2.1. Phosphatidylcholine Species of Controls and Patients with SIRS/Sepsis

This study assessed 21 PC species in the plasma of 160 patients diagnosed with SIRS (n = 38), sepsis (n = 41), or septic shock (n = 81) (Table 1), as well as 23 healthy controls. The patient cohort comprised 49 females and 111 males, whereas the control group comprised 13 females and 10 males (*p* = 0.019). Age of the controls was 42 (25–78) years and differed significantly between patients and controls (*p* < 0.001).

Comparison of PCs between controls and patients with SIRS/sepsis showed that PC 32:2, 34:2, 34:3, 36:2, 36:3, 36:4, 36:5, 38:3, 38:4, 38:5, 38:6, 38:7, 40:4, 40:5, 40:7, and total PC levels were lower in patients’ plasma. However, PC 32:0 was increased (Table 2).

### 2.2. Phosphatidylcholine Species of Patients with SIRS/Sepsis and Liver Cirrhosis

Liver cirrhosis is associated with reduced levels of various lipid classes in blood [19], and PC 34:1, 34:2, 34:3, 36:4, 36:5, 38:3, 38:4, 38:5, 38:6, 40:5, 40:6, 40:7, and total PC levels were reduced. Again, PC 32:0 was higher in the plasma of patients with cirrhosis (Table 3).

The hepatic PC/phosphatidylethanolamine (PE) ratio is associated with liver health [23]. However, total PE levels were also reduced in cirrhosis, and the plasma PC/PE ratio did not change (Table 3). Indeed, the respective PE species analyzed declined in parallel with the PC species (Table 3).

The area under the receiver operating characteristic curve (AUROC) for PC 32:0 was 0.677 (*p* = 0.001) for discriminating between SIRS/sepsis patients with and without cirrhosis. A level of 21.1 nmol/mL predicted cirrhosis with 68% sensitivity and 69% specificity. The AUROCs were lower for all other PC and PE species, total PC and PE levels, and the PC/PE ratio.

The levels of saturated PC species tended to be higher in patients with cirrhosis (*p* = 0.065) than in those without. Those PC species with one to seven double bonds decreased (*p* = 0.016, 0.001, 0.013, <0.001, <0.001, <0.001, and 0.007, respectively).

### 2.3. Phosphatidylcholine and Phosphatidylethanolamine Species of Controls and Patients with SIRS/Sepsis, Excluding Those with Liver Cirrhosis

When SIRS/sepsis patients with liver cirrhosis were excluded, a comparison showed that the levels of PC 32:2, 34:2, 34:3, 36:2, 36:3, 36:4, 36:5, 38:3, 38:4, 38:5, 38:6, 38:7, 40:4, 40:5, and 40:7, as well as total PC levels, were reduced in the patients compared to the healthy controls. PC 32:0 levels were higher in the plasma of patients with SIRS/sepsis than in controls (Table 4).

Multivariate linear regression in patients and controls revealed that sex and age did not predict PC 32:0 (*p* = 0.228), PC 32:1 (*p* = 0.050), PC 34:1 (*p* = 0.339), PC 34:2 (*p* = 0.184), PC 34:3 (*p* = 0.113), PC 36:4 (*p* = 0.549), PC 36:5 (*p* = 0.931), PC 38:4 (*p* = 0.168), PC 38:5 (*p* = 0.362), PC 38:6 (*p* = 0.736), PC 40:5 (*p* = 0.329), PC 40:6 (*p* = 0.934), and PC 40:7 (*p* = 0.584).

PC species predicted by age but not sex were: PC 30:0 (*p* = 0.030 with age being significant, *p* = 0.015), PC 32:2 (*p* = 0.004 with age being significant, *p* = 0.001), PC 36:1 (*p* = 0.044 with age being significant, *p* = 0.027), PC 36:2 (*p* < 0.001, with age being significant, *p* < 0.001), PC 36:3 (*p* = 0.004, with age being significant, *p* = 0.001), PC 38:3 (*p* = 0.010, with age being significant, *p* = 0.003), PC 38:7 (*p* = 0.015, with age being significant, *p* = 0.005), and PC 40:4 (*p* = 0.024, with age being significant, *p* = 0.006).

Patients were older than the controls, so an age-matched comparison was also performed. In patients without cirrhosis aged <62 years (66 patients), age did not significantly differ from that of the 20 controls (*p* = 0.068). However, sex differed significantly between both cohorts (*p* = 0.004). In these subgroups, PC 32:2 (*p* < 0.001), 34:3 (*p* = 0.007), 36:3 (*p* = 0.002), 36:5 (*p* = 0.008), 38:3 (*p* = 0.004), 38:5 (<0.001), 38:6 (*p* = 0.020), 38:7 (*p* = 0.002), 40:4 (*p* < 0.001), and 40:7 (*p* = 0.04) were lower in SIRS/sepsis than in the controls. This shows that PC 32:2, 36:3, 38:3 and 40:4, which were predicted by age, were still reduced in SIRS/sepsis compared to controls when matched for age. Total PC levels were not different between controls and patients with SIRS/sepsis. Again, PC 32:0 (*p* = 0.003) was higher in the plasma of patients. The PC/PE ratio of patients was reduced (*p* < 0.001).

Sex did not predict PC species levels in our cohort. Given the female predominance in the control cohort, a sex-specific analysis was nevertheless conducted. Age differed significantly between female patients without cirrhosis and controls (*p* = 0.005). PC 32:2 (*p* < 0.001), 34:3 (*p* = 0.001), 36:2 (*p* = 0.002), 36:3 (*p* < 0.001), 36:5 (*p* = 0.04), 38:3 (*p* < 0.001), 38:5 (*p* = 0.002), 38:7 (*p* = 0.004), 40:4 (*p* < 0.001), and 40:7 (*p* = 0.010) were lower in female patients compared to female controls. PC 32:0 and total PC levels were not different between controls and patients with SIRS/sepsis. The PC/PE ratio was reduced in patients (*p* = 0.004). In male patients without cirrhosis age (*p* = 0.007) was higher and PC 32:2 (*p* = 0.008), 36:3 (*p* = 0.015), 38:3 (*p* = 0.032), 38:5 (*p* = 0.005), 38:7 (*p* = 0.020), and 40:4 (*p* = 0.017) were reduced compared to controls. PC 32:0 and total PC levels were not different between controls and patients with SIRS/sepsis. The PC/PE ratio was reduced in patients (*p* = 0.002). The sex-specific subcohorts were small, and this may limit statistical power.

### 2.4. Phosphatidylcholine and Phosphatidylethanolamine Species of Patients with SIRS/Sepsis

Since phosphatidylethanolamine N-methyltransferase (PEMT) converts PE to PC, it is important to examine the corresponding PE levels. However, while PC 32:1 and PC 34:1 remained unchanged in cases of SIRS/sepsis, PE 32:1 and PE 34:1 increased significantly. PC 34:2 and PC 38:6 decreased, while PE 34:2 and PE 38:6 increased in the patients’ plasma. In SIRS/sepsis, the total PC levels were low, whereas the total PE levels remained unchanged (Table 4).

**Figure 1:**
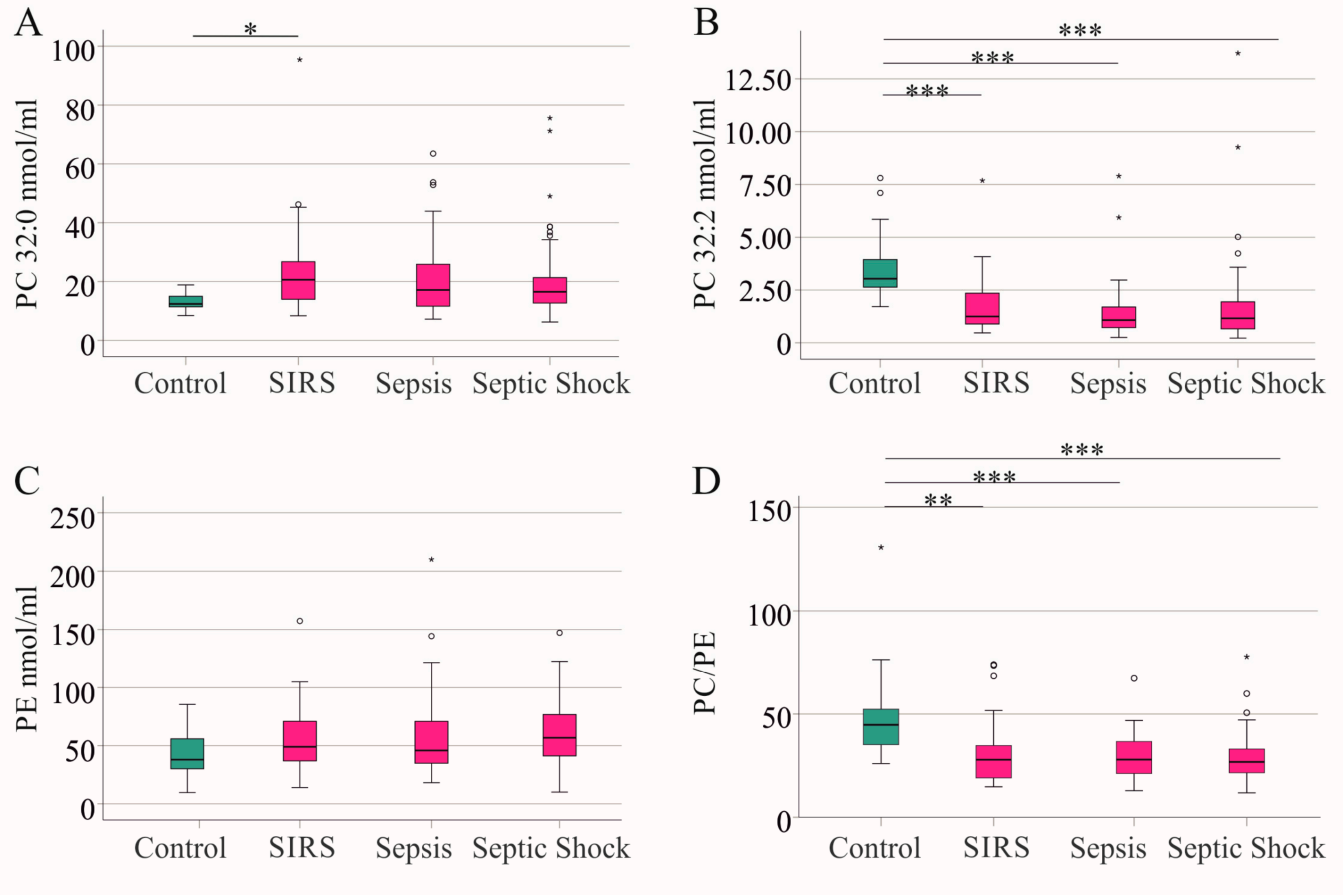
Phosphatidylcholine (PC) and phosphatidylethanolamine (PE) levels of controls and patients (excluding patients with cirrhosis). (**A**) PC32:0 levels in plasma of controls, patients with systemic inflammatory response syndrome (SIRS), sepsis, or septic shock; (**B**) PC 32:2 levels in plasma of controls, patients with SIRS, sepsis, or septic shock; (**C**) Total PE levels in plasma of controls, patients with SIRS, sepsis, or septic shock; (**D**) PC/PE ratio of controls, patients with SIRS, sepsis, or septic shock. * *p* < 0.05, ** *p* < 0.01, *** *p* < 0.001. Small circles and asterisk are outliers.

The PC species levels in patients with SIRS, sepsis, or septic shock were similar (Figure 1A,B; p > 0.05 for all). Total PE levels did not differ between these groups (Figure 1C). The PC/PE ratio was comparably reduced in patients with SIRS, sepsis, or septic shock (Figure 1D).

**Figure 2:**
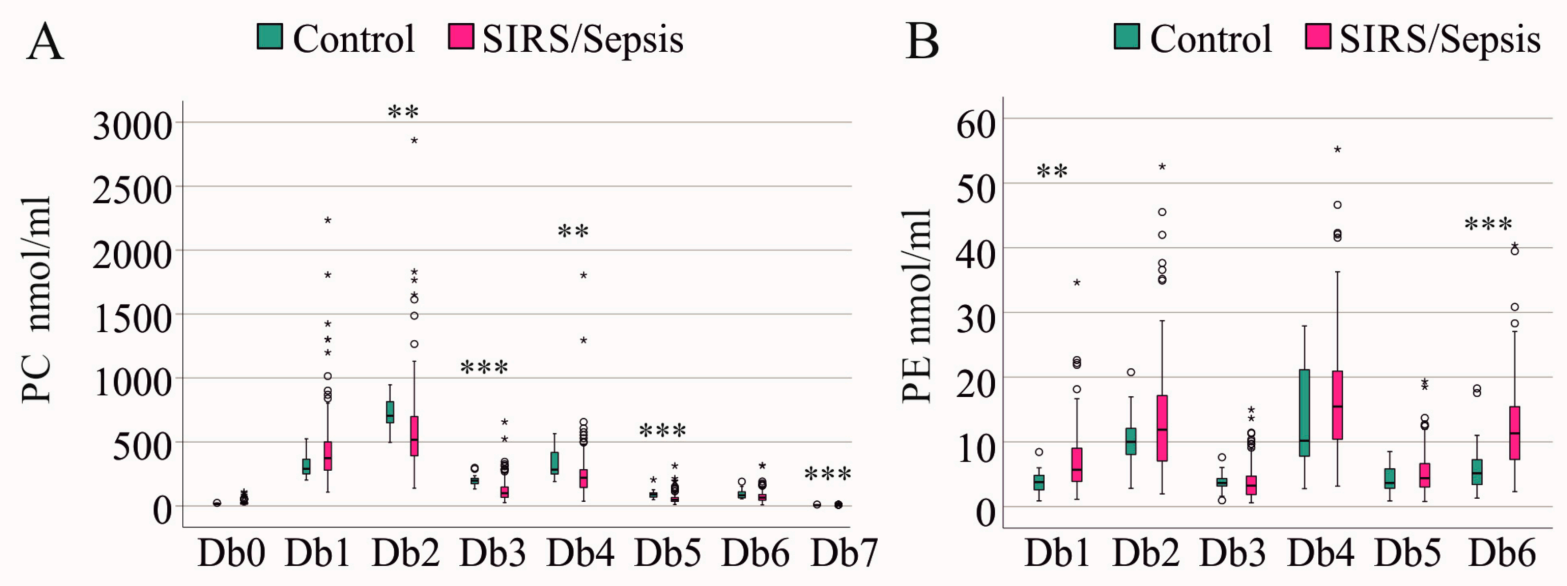
Phosphatidylcholine (PC) and phosphatidylethanolamine (PE) levels categorized by the number of double bonds (Db) of controls and patients. (**A**) PC species with 0–7 Dbs of patients and controls; (**B**) PE species with 1–6 Dbs of patients and controls. ** *p* < 0.01, *** *p* < 0.001. Small circles and asterisk are outliers.

In SIRS/sepsis, there was a significant reduction in the amount of polyunsaturated PCs with two or more double bonds, except for those with six double bonds. Saturated and monounsaturated species remained unchanged (Figure 2A). Regarding PE species, those with one or six double bonds increased significantly. No significant differences were observed in the levels of PC and PE species with the same number of double bonds among patients with SIRS, sepsis, and septic shock. (*p* > 0.05).

### 2.5. Correlation and Multiple Linear Regression Analysis

In SIRS/sepsis patients without cirrhosis, the PC species and PC/PE ratio were not associated with body mass index, leukocyte count, or the number of neutrophils, basophils, eosinophils, monocytes, lymphocytes, or immature granulocytes. Albumin levels, glomerular filtration rate, and creatinine were not related to PC species levels. However, PC 30:0 (r = 0.453, *p* < 0.001), PC 32:0 (r = 0.374, *p* < 0.001), PC 32:1 (r = 0.372, *p* < 0.001), PC 32:2 (r = 0.345, *p* = 0.002), and PC 38:7 (r = 0.307, *p* = 0.012) positively correlated with procalcitonin. PC 34:1 (r = 0.271, *p* = 0.045) and PC 34:2 (r = 0.326, *p* = 0.004) positively correlated with C-reactive protein.

Most PC species with shorter acyl chains (C30-C32) correlated with aspartate and alanine aminotransferase (AST, ALT) (Table 5). Eight PC species correlated with bilirubin and 13 with gamma-glutamyltransferase (GGT). All but PC 30:0, 32:0, 32:1, and 38:7 correlated with plasma cholesterol levels (Table 5). The PC/PE ratio was modestly and positively associated with plasma cholesterol levels (Table 5).

Correlation analysis suggests that higher bilirubin in patients with liver cirrhosis may contribute to increased PC 32:0 levels in these patients (Table 5). In the entire patient cohort, multiple linear regression analysis showed that PC 32:0 is predicted by bilirubin, cholesterol, albumin, and GGT but not by sex, cirrhosis, BMI, creatinine, GFR, AST, or ALT (Table S1). All PC species were significantly predicted by total cholesterol levels (Table S1). PC 30:0, 32:1, 32:2, 36:1, 36:2 and 38:7 were also predicted by bilirubin, and PC 32:1 and 36:1 by albumin (Table S1). PC 36:3 was the only species significantly predicted by sex (Table S1).

Excluding patients with liver cirrhosis revealed that sex, age, BMI, bilirubin, albumin, AST, ALT, GGT, creatinine, and GFR did not predict PC 30:0 (*p* = 0.051), PC 32:1 (*p* = 0.246), PC 32:2 (*p* = 0.517), PC 34:1 (*p* = 0.870), PC 34:2 (*p* = 0.976), PC 34:3 (0.809), PC 36:1 (*p* = 0.709), 36:2 (*p* = 0.697), PC 36:3 (*p* = 0.841), PC 36:4 (*p* = 0.941), PC 36:5 (*p* = 0.942), PC 38:3 (*p* = 0.855), PC 38:4 (*p* = 0.967), PC 38:5 (*p* = 0.859), PC 38:6 (*p* = 0.699), PC 40:4 (*p* = 0.956), PC 40:5 (*p* = 0.853), PC 40:6 (*p* = 0.717), and PC 40:7 (*p* = 0.837).

PC 32:0 was predicted by sex, age, BMI, bilirubin (*p* < 0.001), albumin, AST (*p* = 0.027), ALT (*p* = 0.047), GGT (*p* = 0.043), creatinine, and GFR (F(10,74) = 2.531, *p* = 0.011). PC 38:7 was predicted by sex, age, BMI, bilirubin (*p* = 0.007), albumin, AST, ALT, GGT (*p* = 0.003), creatinine, and GFR (F(10,74) = 2.358, *p* = 0.018).

### 2.6. Phosphatidylcholine Species of Patients with SIRS/Sepsis and Underlying Cholangitis or SARS-CoV-2 Infection

**Figure 3:**
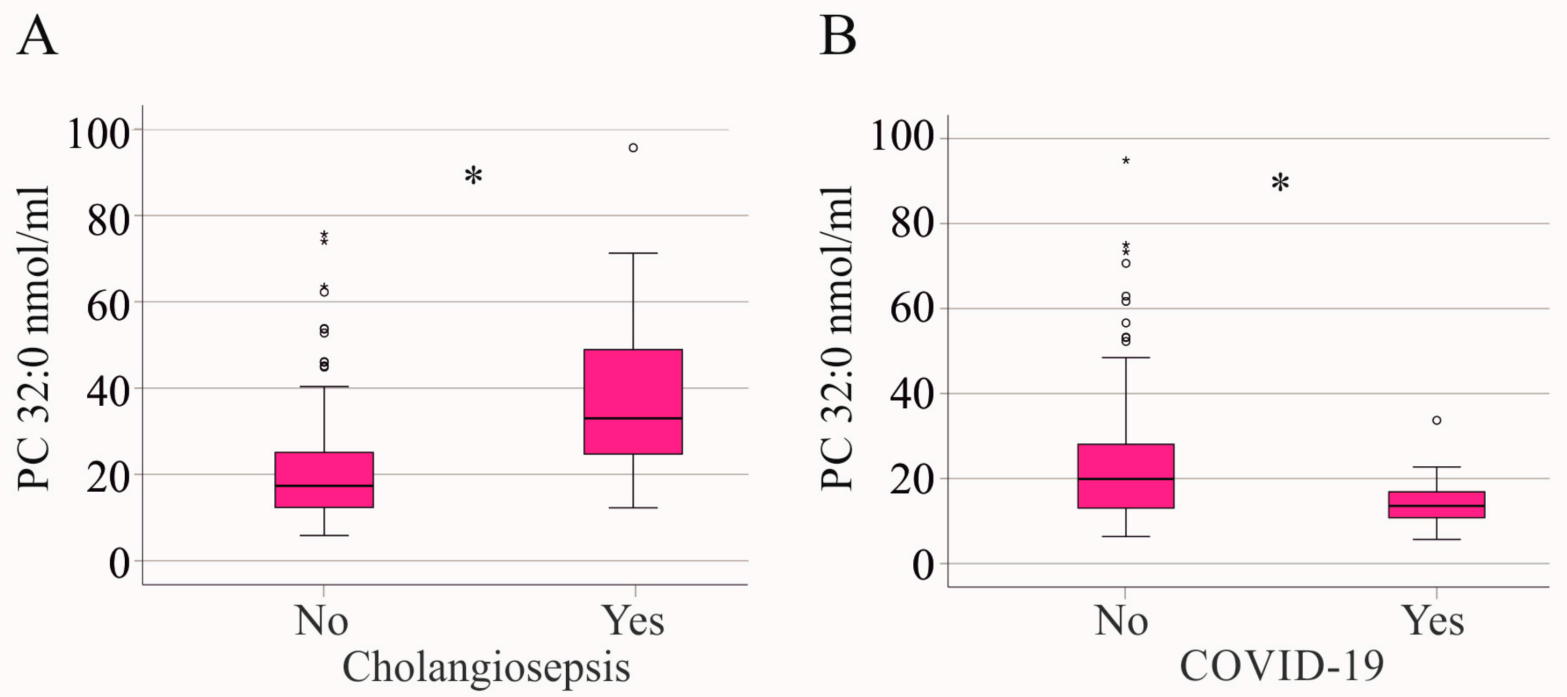
Phosphatidylcholine (PC) 32:0 in patients with cholangiosepsis or COVID-19. Patients with liver cirrhosis were excluded. (**A**) PC 32:0 in plasma of patients with (Yes) and without (No) cholangiosepsis; (**B**) PC 32:0 in plasma of patients with (Yes) and without (No) COVID-19. * *p* < 0.05. Small circles and asterisk are outliers.

Patients with pancreatitis (32 patients) had similar levels of all PC species and PC/PE ratios as those without pancreatitis. Nine patients with cholangiosepsis had higher levels of PC 32:0 (*p* = 0.046; Figure 3A) and PC 32:2 (*p* = 0.032) than patients without cholangiosepsis. PE species did not differ among patients with cholangiosepsis (*p* > 0.05 for all comparisons).

Patients with COVID-19 (22 patients) had lower levels of PC 32:0 (*p* = 0.039; Figure 3B), although no differences were observed in other PC or PE species or in the PC/PE ratio.

The bilirubin and GGT levels of patients with and without cholangiosepsis and/or COVID-19 did not differ significantly, ruling out these factors as the for the cause of altered PC species levels.

### 2.7. Phosphatidylcholine Species in Relation to Interventions and Survival

Dialysis (38 patients), ventilation (77 patients), and vasopressor therapy (75 patients) did not alter plasma PC species levels or the PC/PE ratio (*p* > 0.05 for all comparisons) in patients with SIRS/sepsis excluding those with cirrhosis.

PC species levels, PE species, and the PC/PE ratio were not related to survival, as survivors and non-survivors (N = 25) had similar levels.

## 3. Discussion

**Figure 4:**
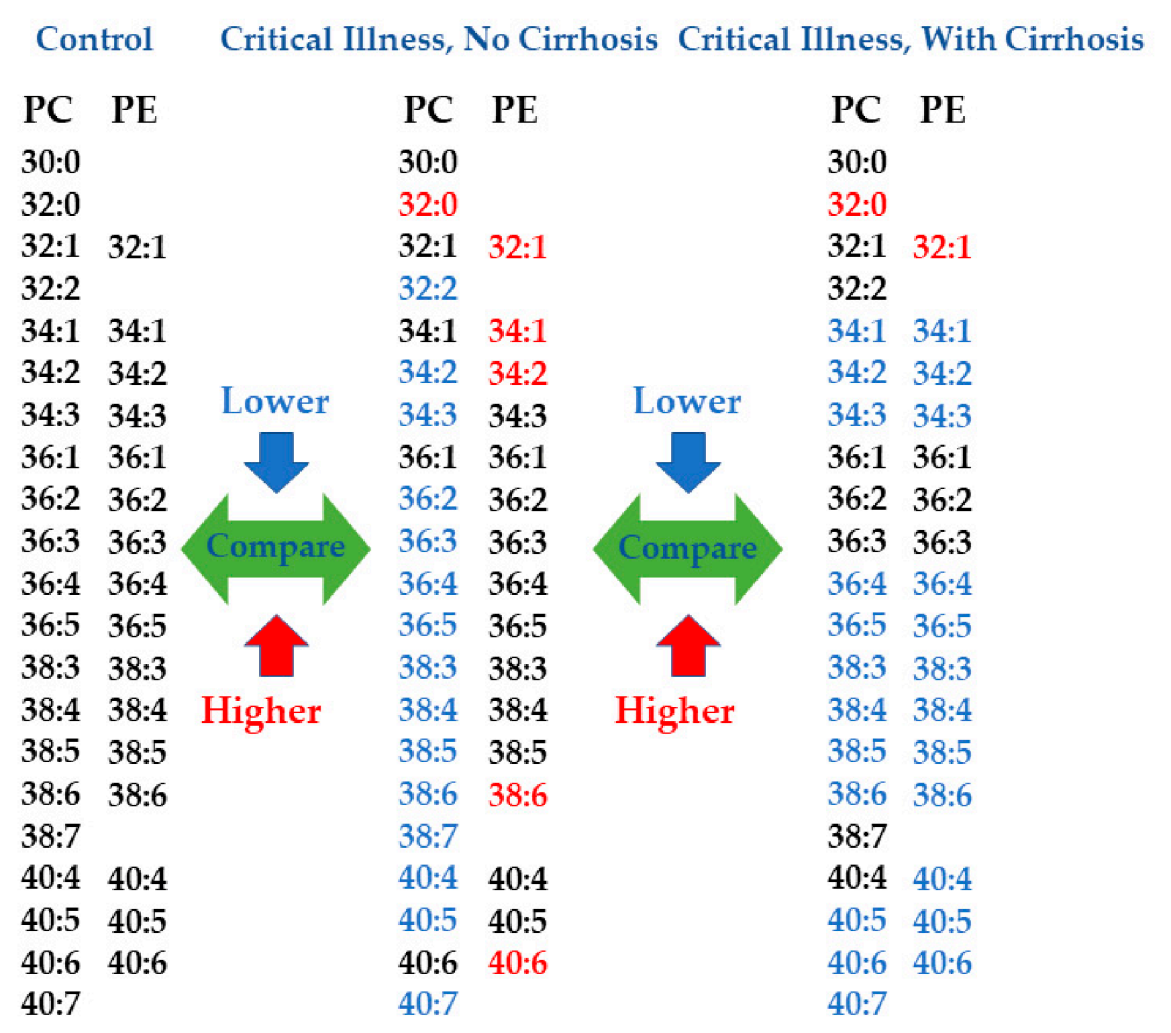
Phosphatidylcholine (PC) and phosphatidylethanolamine (PE) in the plasma of patients with SIRS/sepsis without liver cirrhosis in comparison to controls and in comparison to SIRS/sepsis patients with cirrhosis.

Our data indicate that sepsis is characterized by broad depletion of circulating PC species, which is aggravated by liver cirrhosis, whereas PC 32:0 is selectively increased in both conditions. Whereas the decline of PC species in SIRS/sepsis is mostly not accompanied by lower levels of PE, PC and PE mostly decline in parallel in patients with SIRS/sepsis and cirrhosis (Figure 4). Notably, PC species levels do not provide diagnostic or prognostic value in patients with SIRS/sepsis.

Lodge et al. reported lower levels of PC 16:0/20:2, PC 18:0/20:3, PC 18:2/20:1, PC 18:2/20:3, and PC 20:0/20:3 in sepsis compared with healthy controls [13]. Because the exact fatty acid composition of the measured PC species was not resolved in our study, direct comparisons are limited. Nevertheless, PC 38:3, for which PC 18:0/20:3 is a major constituent [31], was reduced in our SIRS/sepsis cohort, consistent with the findings of Lodge et al. [13]. Overall, both studies support the concept that most of the unsaturated PC species decline in sepsis.

By contrast, Mecatti et al. reported higher levels of PC 15:0/18:2, PC 16:0/18:2, PC 16:0/18:1, and PC 16:0/20:5, along with lower levels of PC 16:0/20:3 and PC 16:0/20:1, in sepsis compared with healthy controls [11]. PC 16:0/18:2 and PC 16:0/18:1 are major contributors to PC 34:2 and PC 34:1, respectively [31]. In our cohort, however, PC 34:2 was modestly reduced, and PC 34:1 was unchanged. These differences underscore the difficulty of comparing studies when lipid species are reported at different levels of structural resolution [13,31]. Larger, more standardized analyses across independent cohorts will be required to determine which PC species are consistently altered in critical illness.

The association between individual PC species and SIRS/sepsis severity also remains uncertain. In our cohort, plasma PC levels did not differ according to dialysis, mechanical ventilation, or vasopressor therapy, and patients with SIRS, sepsis, and septic shock showed largely comparable PC profiles. In contrast, Lodge et al. reported a further decline of several PC species, including PC 18:2/20:3, PC 20:0/20:3, and PC 18:0/20:5, in septic shock compared with sepsis [13]. Thus, current data do not yet establish a consistent relationship between circulating PC species, disease severity, and outcome.

Previous studies did not examine PC species in relation to survival in sepsis. In our cohort, plasma PC levels were similar in intensive care unit (ICU) survivors and non-survivors. A two-sample Mendelian randomization study suggested that PC 16:0/18:2, PC 18:0/18:1, and PC 18:0/20:4 were associated with lower 28-day mortality, whereas PC 16:1/20:4 was associated with higher mortality [32]. Together, these findings indicate that the link between individual PC species and survival remains unresolved.

Experimental evidence supports a possible immunomodulatory role, as PC supplementation reduced lipopolysaccharide-induced inflammation in mice [9,33]. Consistent with the concept that limiting excessive inflammation may improve outcomes, cytokine inhibitors have been reported to reduce sepsis-related mortality by 12% [34]. However, PC supplementation has not, to our knowledge, been tested in patients with sepsis.

Evidence on circulating PC species is more consistent in liver cirrhosis than in sepsis. Patients with chronic hepatitis C and cirrhosis showed increased serum PC 30:0 and PC 32:0, whereas polyunsaturated PC species were reduced [20]. Similar reductions in polyunsaturated PCs were reported in chronic hepatitis B virus infection [21]. Consistent with these observations, our patients with SIRS/sepsis and cirrhosis also exhibited lower polyunsaturated PC species and higher PC 32:0 levels.

Accordingly, SIRS/sepsis and cirrhosis shared a common pattern of depletion affecting most polyunsaturated PC species. Ten of the 21 measured PC species were reduced in both conditions. However, PC 32:2, PC 36:2, PC 36:3, PC 38:7, and PC 40:4 decreased only in SIRS/sepsis, whereas PC 34:1 decreased only in cirrhosis, supporting disease-specific regulation of individual PC species. Corresponding PE species declined in cirrhosis but not in sepsis, suggesting that reduced PC levels in cirrhosis reflect broader alterations in PE and systemic lipid homeostasis [19]. By contrast, the mechanisms driving PC remodeling in sepsis remain poorly defined.

Patients with SIRS/sepsis and cirrhosis showed the most pronounced reduction in plasma PC levels, whereas PC 32:0 was selectively increased in both conditions. PC 32:0 levels were 38% higher in patients with SIRS/sepsis (excluding those with cirrhosis) than in healthy controls. Among patients with SIRS/sepsis, those with cirrhosis exhibited PC 32:0 levels approximately 55% of those observed in patients without cirrhosis. This is comparable to the 52% increase reported in patients with HCV-associated cirrhosis relative to those without cirrhosis at the end of antiviral therapy [20]. Similarly, Chen et al. reported a non-significant increase in PC 32:0 concentrations from approximately 16 µmol/L to 22 µmol/L (about 38%) in patients with HBV-associated cirrhosis compared with those without cirrhosis [21]. Together, these findings suggest that circulating PC 32:0 levels are elevated in cirrhosis independently of the presence of SIRS/sepsis. This should be confirmed by including a cirrhosis-only cohort in the lipid analysis. PC 32:0 is thought to consist predominantly of PC 16:0/16:0 [31], but this species was not reported as altered in previous sepsis studies and may not have been specifically measured [11,13]. In our cohort, PC 32:0 showed a positive correlation with bilirubin, suggesting that cholestatic or hepatobiliary dysfunction may contribute to its elevation. Patients with cholangiosepsis also had higher PC 32:0 levels, which is consistent with this interpretation, although bilirubin was not significantly elevated in this subgroup.

Most unsaturated PC species showed positive correlations with cholesterol, which is typically reduced in both cirrhosis and sepsis [6,19,29,35], and this may contribute to the lower plasma PC levels observed in both conditions. In contrast, PC 32:0 did not correlate with cholesterol, suggesting that its regulation is distinct from that of most other PC species. However, multiple linear regression analysis of the entire SIRS/sepsis cohort revealed that total cholesterol levels were significant predictors of all PC species, indicating that the elevated PC 32:0 levels are also associated with disturbed cholesterol homeostasis in SIRS/sepsis. In addition, PC 30:0, 32:1, 32:2, 36:1, 36:2 and 38:7 were significantly associated with bilirubin levels, whereas PC 32:1 and 36:1 were predicted by albumin, suggesting that these species are more closely linked to hepatic function. It should be noted that the plasma levels of these latter PC species—except PC 32:0—did not change in cirrhosis and may be related to altered bilirubin metabolism in SIRS/sepsis, which can be caused by increased hemolysis as well as liver dysfunction [36]. In the entire cohort, PC 36:3 was the only species significantly predicted by sex (Table S1). Plasma PC 36:3 levels were similar in both sexes in the entire cohort and in cirrhosis and the association of sex with this species remains unclear. When excluding patients with cirrhosis, most PC species were not predicted by sex, age, BMI, bilirubin, albumin, AST, ALT, GGT, creatinine, and GFR. PC 32:0 was predicted by bilirubin, AST, ALT, and GGT, and PC 38:7 by bilirubin and GGT. Taken together, these analyses indicate that, except for cholesterol, the factors responsible for altered PC levels in SIRS/sepsis remain largely unknown.

Sex and age differed between the control group and patients with SIRS/sepsis. Age was a significant predictor of PC 30:0, 32:2, 36:1, 36:2, 36:3, 38:3, 38:7 and 40:4. To account for this potential confounding effect, PC species levels were compared in age-matched subgroups. This analysis showed that PC 32:0 remained significantly increased in patients, whereas ten PC species (32:2, 34:3, 36:3, 36:5, 38:3, 38:5, 38:6, 38:7, 40:4, and 40:7) remained significantly decreased. These findings indicate that the observed differences in PC species levels between patients and controls cannot be explained solely by differences in age. The increase in PC 32:0 could not be confirmed in sex-stratified analyses, whereas PC 32:2, 36:3, 38:3, 38:5, 38:7, and 40:4 were consistently decreased in both male and female patients. In contrast, decreases in PC 34:3, 36:2, 36:5, and 40:7 were observed only in female patients with SIRS/sepsis compared with female controls; therefore, a sex-specific effect cannot be excluded. However, the small size of these sex-stratified cohorts limits statistical power, and analyses in larger cohorts are required to determine whether these differences truly reflect sex-specific alterations.

PC species with shorter acyl chains showed positive correlations with C-reactive protein and procalcitonin. Because PC 32:0 was the only PC species consistently increased in SIRS/sepsis, its relationship to systemic inflammation deserves further investigation.

The mechanisms underlying the selective increase in PC 32:0 in sepsis, and the lower levels of PC 32:0 in SARS-CoV-2-associated sepsis compared with other sepsis etiologies, remain unclear. One possible explanation is increased availability of palmitate, as fatty acid synthase is upregulated in sepsis [37]. This would be consistent with higher PC 32:0 concentrations, given that PC 32:0 is thought to be composed predominantly of PC 16:0/16:0 [31]. However, this will not explain lower levels in COVID-19. Moreover, PC biosynthesis is regulated by multiple enzymatic pathways, and this hypothesis requires further study.

PEMT catalyzes the conversion of PE to PC [25]. However, lower levels of PC 36:2, PC 36:3, and PC 36:4 were observed in SIRS/sepsis, despite unchanged levels of the corresponding PE species. These findings do not support a reduced PEMT activity in critical illness as an explanation for the decline in predominantly unsaturated PC species. However, PC levels in blood are affected by multiple mechanisms and pathways [8,23,38], and this observational study cannot exclude a role of PEMT in PC remodeling in SIRS/sepsis.

The hepatic PC/PE ratio is considered a marker of liver integrity [25]. In plasma, however, the PC/PE ratio was similar in patients with sepsis with and without cirrhosis. In SIRS/sepsis, the ratio decreased primarily because PC levels declined, whereas PE levels remained largely unchanged. The ratio was not associated with inflammatory markers or indices of liver disease and correlated only modestly with cholesterol. It may therefore reflect a distinct aspect of systemic lipid remodeling and warrants further evaluation as a potential biomarker in sepsis.

Strengths of our study include the relatively large SIRS/sepsis cohort and the single-center design, which likely improved procedural consistency. However, the single-center setting may limit generalizability and highlight the need for external multicenter validation. Additional limitations include non-fasting sampling conditions and the availability of only a single blood sample obtained during the early phase of illness. The relatively small healthy control group may have introduced selection bias. The controls were younger and included more females and this mismatch limits interpretation of the data. A small control group may not adequately reflect the heterogeneity of the general population. Among individuals aged approximately 60 years, chronic conditions such as hypertension, cardiovascular disease, and type 2 diabetes are common, with more than 50% experiencing multimorbidity [39]. However, individuals with these conditions were excluded from the control group. Accounting for the full spectrum of comorbidities would require substantially larger control cohorts. Furthermore, patients with liver cirrhosis in the absence of critical illness were not included, preventing comparisons between patients with cirrhosis with and without SIRS or sepsis.

## 4. Materials and Methods

### 4.1. Study Cohort

Blood samples were obtained 12–24 h after admission from patients treated in the medical ICU of a German university hospital between August 2018 and January 2024. Plasma samples from patients with COVID-19 were collected between October 2020 and January 2023. Patients were categorized as having SIRS, sepsis, or septic shock according to established SIRS and Sepsis-3 criteria [40,41]. Exclusion criteria were multidrug-resistant infections, hepatitis virus infection, and human immunodeficiency virus infection. All eligible patients who provided informed consent were included. ICU mortality was used to define outcome: patients who died during the ICU stay were classified as non-survivors, and those discharged alive as survivors. Routine laboratory parameters and microbiological findings were obtained from the respective institutional diagnostic laboratories of University Hospital Regensburg.

The control group consisted of 23 healthy volunteers (10 men and 13 women; median age 42 years, range 25–78) recruited from hospital staff, students, and their relatives. All controls had a normal body weight. Laboratory analyses were not performed in this group.

### 4.2. Measurement of Plasma PC Species

PC species were quantified by flow injection analysis–Fourier transform mass spectrometry (FIA-FTMS) using a hybrid quadrupole–Orbitrap instrument (Thermo Fisher Scientific, Waltham, MA, USA). The analytical workflow has been described in detail previously [42]. Prior to lipid extraction, internal standards PC 14:0/14:0 (1.29 nmol) and PC 22:0/22:0 (1.39 nmol) were added to each sample. Plasma aliquots (10 µL) were extracted according to the method of Bligh and Dyer [43]. Following phase separation, the chloroform (lipid-containing) fraction was transferred using a Tecan Genesis RSP 150 pipetting robot (Tecan Schweiz AG, Männedorf, Zürich, Switzerland) and then evaporated to dryness under reduced pressure. The dried extract was reconstituted in chloroform/methanol/2-propanol (1:2:4, *v*/*v*/*v*) supplemented with 7.5 mM ammonium formate. PC species were detected as formate adducts [M + HCOO]^−^ in negative ion mode across an *m*/*z* range of 520–960. Instrument parameters were as follows: maximum injection time 200 ms, automated gain control (AGC) target 1 × 10^6^, three microscans, and a resolving power of 140,000 at *m*/*z* 200 [44]. Quantification was achieved by multiplying the known amount of the spiked internal standard by the analyte-to-internal standard peak area ratio. The designation PC 32:1 indicates a phosphatidylcholine species comprising one saturated and one monounsaturated fatty acyl chain with a total carbon number of 32. This analytical configuration does not permit the determination of fatty acyl composition or positional isomerism. Analysis of PEs has been described recently [45]. Original data are shown in Table S2.

### 4.3. Statistical Analysis

PC species were not normally distributed (Shapiro–Wilk test, *p* < 0.001 for all), and data are shown as box plots with the median, first and third quartiles, and minimum and maximum values displayed. Asterisks or circles indicate outliers. Median, minimum, and maximum values are also given in tabular form. Statistical analyses were performed using IBM SPSS Statistics 31.0.0.0 (IBM Corp., Armonk, NY, USA; released 2019, updated 2025). The tests used to assess relationships between variables were:

1. Chi-squared test for categorical variables;
2. Mann–Whitney U test for comparisons between two groups;
3. Kruskal–Wallis test for comparison of three or more groups;
4. Spearman’s correlation for associations between continuous variables;
5. Receiver operating characteristic (ROC) curve analysis.
6. Multiple linear regression

A two-sided *p* value < 0.05 was considered statistically significant. For the analysis of PC and PE species, *p* values were adjusted for multiple comparisons using the Bonferroni method by multiplying each *p* value by 21, corresponding to the number of PC species analyzed. No adjustment for multiple comparisons was applied to any other analyses.

## 5. Conclusions

In conclusion, SIRS/sepsis was characterized by broad depletion of circulating PC species, particularly among unsaturated PCs, and this pattern was further accentuated by underlying liver cirrhosis. By contrast, PC 32:0 was consistently increased in both SIRS/sepsis and SIRS/sepsis cirrhosis, identifying it as a distinctive feature of PC remodeling in critical illness. These findings suggest that individual PC species are differentially regulated rather than uniformly reduced and highlight PC 32:0 as a candidate for further mechanistic investigation.

## Figures and Tables

**Table 1:** Details of the patient cohort: Values are reported as median (minimum–maximum). A superscript number was used when the respective value was unavailable for the entire cohort.

Parameters	SIRS/Sepsis/Septic Shock Patients (N = 160)
Males/Females	111/49
Age, years	59 (21–93)
Body mass index, kg/m^2^	26.2 (15.4–55.6) ^155^
SIRS/Sepsis/Septic shock	38/41/81
C-reactive protein, mg/L	158 (12–697) ^157^
Procalcitonin, ng/mL	1.13 (0.05–270.00) ^154^
Leukocytes, n/nL	10.32 (0.06–1586.00) ^157^
Neutrophils, n/nL	7.72 (0–70.20) ^152^
Basophils, n/nL	0.04 (0–0.90) ^153^
Eosinophils, n/nL	0.13 (0–8.80) ^153^
Monocytes, n/nL	0.78 (0–45.00) ^153^
Lymphocytes, n/nL	0.94 (0.08–28.60) ^153^
Immature granulocytes, n/nL	0.12 (0–6.19) ^151^
Total bilirubin, mg/dL	1.00 (0.10–36.60) ^150^
Albumin, g/L	22.4 (6.3–42.0) ^146^
Aspartate aminotransferase, U/L	46 (6–1597) ^143^
Alanine aminotransferase, U/L	32 (6–770) ^141^
Gamma glutamyltransferase, U/L	115 (11–1266) ^127^
Glomerular filtration rate mL/min	52 (6–140) ^143^
Creatinine mg/dL	1.40 (0.33–7.66) ^143^

**Table 2:** The median (minimum–maximum) plasma PC levels are shown for the control and the SIRS/sepsis groups. The arrows indicate a decline ⇓ or increase ⇑ in patients with SIRS/sepsis compared with controls.

PC nmol/mL	Control (N = 23)	SIRS/Sepsis (N = 160)	*p*-Value
30:0	2.8 (1.6–7.2)	2.1 (0.6–24.8)	>0.05
32:0	12.4 (8.4–18.8)	18.8 (6.2–95.5)	0.002 ⇑
32:1	15.0 (7.0–56.3)	20.2 (6.0–232.8)	>0.05
32:2	3.0 (1.7–7.8)	1.1 (0–13.7)	<0.001 ⇓
34:1	237.6 (157.9–417.3)	293.9 (78.0–1941.9)	>0.05
34:2	442.9 (324.2–667.8)	335.3 (55.0–1882.4)	0.002 ⇓
34:3	14.3 (10.0–32.7)	7.5 (1.4–80.2)	<0.001 ⇓
36:1	36.5 (24.4–73.4)	35.7 (7.3–173.5)	>0.05
36:2	247.4 (170.9–352.3)	161.9 (35.3–968.8)	<0.001 ⇓
36:3	139.6 (96.0–216.7)	65.5 (13.9–490.6)	<0.001 ⇓
36:4	185.6 (123.5–407.4)	120.1 (9.0–1273.5)	<0.001 ⇓
36:5	20.6 (9.9–88.2)	10.0 (0.8–116.2)	<0.001 ⇓
38:3	38.9 (25.9–90.3)	17.4 (3.6–130.6)	<0.001 ⇓
38:4	99.9 (63.8–179.6)	67.9 (7.6–524.7)	<0.001 ⇓
38:5	56.3 (30.1–101.9)	26.0 (3.5–176.2)	<0.001 ⇓
38:6	60.2 (41.7–147.3)	41.3 (1.7–251.0)	0.001 ⇓
38:7	0.9 (0.3–2.0)	0.4 (0–2.4)	<0.001 ⇓
40:4	3.1 (2.1–7.9)	1.8 (0.2–8.7)	<0.001 ⇓
40:5	9.3 (5.4–19.3)	6.1 (0.8–28.4)	<0.001 ⇓
40:6	21.0 (13.8–42.6)	16.1 (0.9–72.7)	>0.05
40:7	4.5 (3.1–8.6)	2.9 (0.3–14.0)	<0.001 ⇓
Total PC	1712.7 (1249.6–2470.9)	1297.1 (314.7–7844.0)	0.001 ⇓

**Table 3:** The median (minimum–maximum) plasma PC and PE levels are shown for SIRS/sepsis patients with and without liver cirrhosis. The arrows indicate a decline ⇓ or increase ⇑ in patients with cirrhosis compared to those without.

PC nmol/mL	No liver Cirrhosis (129)	Liver Cirrhosis (31)	*p*-Value	PE nmol/mL	No Liver Cirrhosis (129)	Liver Cirrhosis (31)	*p*-Value
30:0	1.9 (0.6–24.8)	2.8 (0.8–9.3)	>0.05				
32:0	17.2 (6.2–95.5)	26.4 (6.9–73.9)	0.047 ⇑				
32:1	21.0 (6.0–232.8)	20.3 (7.2–89.1)	>0.05	32:1	0.2 (0–1.2)	0.1 (0–0.6)	>0.05
32:2	1.1 (0.2–13.7)	0.8 (0–3.6)	>0.05				
34:1	302.3 (92.0–1941.9)	203.0 (78.0–508.1)	0.002 ⇓	34:1	3.2 (0.7–14.6)	1.8 (0.5–8.9)	0.003 ⇓
34:2	350.1 (96.7–1882.4)	196.4 (55.0–467.5)	<0.001 ⇓	34:2	3.8 (0.9–17.7)	1.9 (0.5–9.3)	0.003 ⇓
34:3	8.0 (2.7–80.1)	4.8 (1.4–14.3)	0.002 ⇓	34:3	0.2 (0.0–0.6)	0.1 (0.0–0.5)	0.020 ⇓
36:1	38.0 (9.0–173.5)	31.3 (7.3–102.4)	>0.05	36:1	2.1 (0.4–18.1)	1.7 (0.3–9.3)	>0.05
36:2	166.8 (41.8–968.8)	125.7 (35.3–302.7)	>0.05	36:2	7.8 (1.0–34.6)	6.1 (1.3–24.5)	>0.05
36:3	69.3 (17.4–490.69	47.5 (13.9–101.8)	>0.05	36:3	2.1 (0.4–8.9)	1.9 (0.4–7.2)	>0.05
36:4	137.3 (18.8–1273.5)	47.5 (9.0–149.7)	<0.001 ⇓	36:4	4.2 (0.8–12.2)	1.6 (0.2–9.00)	<0.001 ⇓
36:5	11.4 (1.3–116.2)	5.8 (0.8–17.1)	<0.001 ⇓	36:5	0.3 (0.1–1.2)	0.1 (0.0–0.6)	0.001 ⇓
				38:1	0.1 (0.0–1.2)	0.1 (0–0.6)	0.020 ⇓
				38:2	0.2 (0.1–0.5)	0.2 (0.1–1.2)	>0.05
38:3	19.1 (5.8–130.6)	13.6 (3.6–23.4)	<0.001 ⇓	38:3	0.8 (0.1–5.6)	0.5 (0.1–1.9)	0.003 ⇓
38:4	76.1 (16.5–524.7)	35.9 (7.6–121.3)	<0.001 ⇓	38:4	11.0 (2.2–42.5)	5.0 (0.6–26.1)	<0.001 ⇓
38:5	27.9 (6.8–176.2)	15.7 (3.5–37.5)	<0.001 ⇓	38:5	3.1 (0.5–14.3)	1.5 (0.3–5.4)	<0.001 ⇓
38:6	45.3 (5.0–251.0)	15.9 (1.7–59.5)	<0.001 ⇓	38:6	6.7 (1.5–22.6)	2.1 (0.2–14.0)	<0.001 ⇓
38:7	0.4 (0–2.4)	0.6 (0.1–1.5)	>0.05				
40:4	1.8 (0.5–8.7)	1.5 (0.2–2.5)	>0.05	40:4	0.2 (0.0–1.8)	0.1 (0.0–0.5)	0.001 ⇓
40:5	6.6 (1.8–28.4)	4.2 (0.8–9.0)	<0.001 ⇓	40:5	1.0 (0.2–5.7)	0.3 (0.1–2.2)	<0.001 ⇓
40:6	18.8 (3.1–72.7)	7.1 (0.9–32.5)	<0.001 ⇓	40:6	4.2 (0.7–17.9)	1.3 (0.0–10.4)	<0.001 ⇓
40:7	3.1 (0.7–14.0)	1.8 (0.3–5.6)	<0.001 ⇓				
Total PC	1370.4 (414.3–7844.0)	880.7 (314.7–1959.7)	<0.001 ⇓	Total PE	53.5 (10.1–210.1)	23.9 (6.0–123.3)	<0.001 ⇓
PC/PE	27.1 (11.9–77.8)	30.4 (10.0–58.2)	>0.05				

**Table 4:** The median (minimum–maximum) plasma PC and PE levels of controls and patients with sepsis, excluding those with liver cirrhosis. The arrows indicate a decline ⇓/increase ⇑ in patients compared to controls.

PC nmol/mL	Control (23)	SIRS/Sepsis (129)	*p*-Value	PE nmol/mL	Control (23)	SIRS/Sepsis (129)	*p*-Value
30:0	2.8 (1.6–7.2)	1.9 (0.6–24.8)	>0.05				
32:0	12.4 (8.4–18.8)	17.1 (6.2–95.5)	0.018 ⇑				
32:1	15.0 (7.0–56.3)	20.0 (6.0–232.8)	>0.05	32:1	0.1 (0–0.7)	0.2 (0–1.2)	<0.001 ⇑
32:2	3.0 (1.7–7.8)	1.1 (0.2–13.7)	<0.001 ⇓				
34:1	237.6 (157.9–417.3)	300.7 (92.0–1941.9)	>0.05	34:1	1.5 (0.3–4.5)	3.2 (0.7–14.6)	<0.001 ⇑
34:2	442.9 (324.2–667.8)	349.7 (96.7–1882.4)	0.034 ⇓	34:2	2.4 (0.9–7.0)	3.8 (0.9–17.7)	0.005 ⇑
34:3	14.3 (10.0–32.7)	8.0 (2.7–80.2)	<0.001 ⇓	34:3	0.1 (0.0–0.6)	0.2 (0.1–0.6)	>0.05
36:1	36.5 (24.4–73.4)	38.1 (9.0–173.5)	*>*0.05	36:1	1.9 (0.4–3.4)	2.1 (0.4–18.1)	>0.05
36:2	247.4 (170.9–352.3)	166.0 (41.8–968.8)	<0.001 ⇓	36:2	7.3 (1.8–13.5)	7.8 (1.0–34.6)	>0.05
36:3	139.6 (96.0–216.7)	68.6 (17.6–490.6)	<0.001 ⇓	36:3	2.7 (0.6–5.0)	2.1 (0.4–8.9)	>0.05
36:4	185.6 (123.5–407.4)	134.8 (18.8–1273.5)	0.009 ⇓	36:4	2.9 (0.9–8.5)	4.2 (0.8–13.2)	>0.05
36:5	20.6 (9.9–88.2)	11.3 (1.3–116.2)	<0.001 ⇓	36:5	0.3 (0.1–1.0)	0.3 (0.1–1.2)	>0.05
				38:1	0.2 (0.1–0.4)	0.1 (0–1.2)	<0.001 ⇓
				38:2	0.2 (0.1–0.5)	0.2 (0.1–1.2)	>0.05
38:3	38.9 (25.9–90.3)	19.0 (5.8–130.6)	<0.001 ⇓	38:3	0.8 (0.3–2.0)	0.9 (0.1–5.6)	>0.05
38:4	99.9 (63.8–179.6)	74.6 (16.5–524.7)	0.006 ⇓	38:4	7.1 (1.8–21.5)	11.0 (2.2–42.5)	>0.05
38:5	56.3 (30.1–101.9)	27.8 (6.8–176.2)	<0.001 ⇓	38:5	2.7 (0.6–6.3)	3.0 (0.5–14.3)	>0.05
38:6	60.2 (41.7–147.3)	45.7 (5.0–251.0)	0.033 ⇓	38:6	3.2 (0.9–12.6)	6.7 (1.5–22.6)	<0.001 ⇑
38:7	0.9 (0.3–2.0)	0.4 (0.0–2.4)	<0.001 ⇓				
40:4	3.1 (2.1–7.9)	1.8 (0.5–8.7)	<0.001 ⇓	40:4	0.2 (0.1–1.3)	0.2 (0.0–1.8)	>0.05
40:5	9.3 (5.4–19.3)	6.5 (1.8–28.4)	0.007 ⇓	40:5	0.7 (0.2–2.0)	1.0 (0.2–5.7)	>0.05
40:6	21.0 (13.8–42.6)	18.8 (3.1–72.7)	>0.05	40:6	1.9 (0.4–5.7)	4.2 (0.7–17.9)	<0.001 ⇑
40:7	4.5 (3.1–8.6)	3.0 (0.7–14.0)	<0.001 ⇓				
Total PC	1712.7 (1249.6–2470.9)	1347.0 (414.3–7844.0)	0.017 ⇓	Total PE	38.0 (9.6–85.4)	53.5 (10.1–210.1)	>0.05
PC/PE	44.8 (26.0–130.7)	27.1 (11.9–77.8)	<0.001 ⇓				

**Table 5:** Spearman correlation coefficients for the association of PC species with bilirubin, aspartate aminotransferase (AST), alanine aminotransferase (ALT), gamma-glutamyltransferase (GGT), and plasma cholesterol in patients with SIRS/sepsis excluding those with cirrhosis. *p* < 0.001 (red boxes), *p* < 0.01 (orange boxes), *p* < 0.05 (light red boxes), and *p* > 0.05 (light blue boxes).

PC Species	Bilirubin	AST	ALT	GGT	Cholesterol
30:0	0.504	0.317	0.286	0.372	0.200
32:0	0.490	0.253	0.236	0.290	0.255
32:1	0.413	0.242	0.296	0.379	0.264
32:2	0.416	0.315	0.319	0.527	0.325
34:1	0.238	0.193	0.194	0.338	0.526
34:2	0.288	0.231	0.233	0.348	0.579
34:3	0.293	0.268	0.260	0.523	0.443
36:1	0.240	0.196	0.184	0.367	0.388
36:2	0.332	0.233	0.205	0.376	0.473
36:3	0.158	0.084	0.122	0.498	0.662
36:4	−0.096	−0.011	0.077	0.153	0.704
36:5	−0.069	0.050	0.110	0.364	0.610
38:3	0.041	0.003	0.078	0.442	0.605
38:4	−0.109	−0.043	0.028	0.154	0.680
38:5	−0.105	−0.008	0.036	0.299	0.783
38:6	−0.141	−0.027	0.029	0.199	0.654
38:7	0.397	0.202	0.192	0.379	0.221
40:4	−0.050	0.012	0.020	0.202	0.651
40:5	−0.055	−0.033	0.017	0.291	0.722
40:6	−0.121	−0.037	0.015	0.261	0.578
40:7	0.029	0.065	0.035	0.346	0.603
Total PC	0.198	0.160	0.183	0.361	0.669
PC/PE	−0.018	−0.051	−0.001	0.123	0.278

## Data Availability

The original contributions presented in the study are included in the article/Supplementary Materials, further inquiries can be directed to the corresponding author.

## Supplementary Materials

The following supporting information can be downloaded at: https://www.mdpi.com/article/10.3390/ijms27156858/s1.

## Abbreviations

The following abbreviations are used in this manuscript:

ALT	Alanine aminotransferase
AST	Aspartate aminotransferase
GGT	Gamma-glutamyl transferase
ICU	Intensive care unit
Interleukin	IL
PC	Phosphatidylcholine
PE	Phosphatidylethanolamine
PEMT	Phosphatidylethanolamine N-methyltransferase
SARS-CoV-2	Severe acute respiratory syndrome coronavirus 2
SIRS	Systemic inflammatory response syndrome
